# Relationship between paraspinal muscle degeneration and the global alignment and proportion (GAP) score: cross-sectional study in middle-aged and older adults

**DOI:** 10.1186/s12891-026-10244-x

**Published:** 2026-07-23

**Authors:** Hao Xue, Wei Dai, Guanzhong Wang, Chengbo Hu, Chengmin Zhang, Fei Luo

**Affiliations:** Department of Orthopaedic, Southwest Hospital, The First Affiliated Hospital of Army Medical University, No. 30 Gaotanyan Zhengjie, Shapingba District, Chongqing, 400038 China

**Keywords:** Paraspinal muscle, GAP score, Degeneration, Spinopelvic parameters

## Abstract

**Background:**

The paraspinal muscle, a crucial component of the stable sagittal balance of the spine, is closely associated with the structural degeneration of ligaments. The global alignment and proportion (GAP) score can predict spinal pelvic ligament degeneration and examine the overall sagittal balance of the spine. This study aimed to determine the correlation between the paraspinal muscles and the GAP score using imaging evaluation and to investigate better methods for evaluating the sagittal balance of the spine compared to spinal pelvic parameters.

**Methods:**

This study enrolled 61 patients aged over 45 years who suffered from chronic lower back pain or leg pain. The functional cross-sectional area (FCSA) and fatty infiltration (FI) of the paraspinal muscle were quantified using ImageJ software. The spinopelvic sagittal parameters were measured. Pearson correlation analysis was used to investigate the correlations between paraspinal muscle measurements and GAP score. The participants were stratified into a proportioned (P) group, a moderately disproportioned (MD) group, and a severely disproportioned (SD) group according to the GAP score. The paraspinal muscles were subsequently compared among the three groups.

**Results:**

A significant negative correlation was observed between the multifidus (MF) FCSA, erector spinae (ES) FCSA, and GAP scores (*r* = − 0.339, − 0.409, *P* < 0.01). Additionally, a significant positive correlation was observed between MF FI, ES FI, and GAP scores (*r* = 0.379, 0.354, *P* < 0.01). The SD group showed significantly greater FI and significantly lower FCSA of the MF and ES than the MD and P groups(*P* < 0.05), with no significant differences between the MD and P groups (*P* > 0.05).

**Conclusions:**

The degree of paraspinal muscle degeneration is strongly associated with the GAP score; therefore, more severe paraspinal muscle degeneration corresponds to a higher GAP score. Moreover, the relative sagittal plane parameters (relative pelvic version, relative lumbar lordosis, and relative spinopelvic alignment) exhibited significant correlation with the paraspinal muscle parameters and were more strongly correlated with the paraspinal muscle parameters than with the actual spinal pelvic parameters. These findings suggest that the relative sagittal plane parameters serve as sensitive indicators for assessing sagittal spine balance.

## Introduction

The lumbar spine’s soft tissues are essential for its stability [1]. These structures primarily comprise the ligamentous structure of the spine and the paraspinal muscles [2]. In 2005, Roussouly et al. proposed that differences in the sagittal alignment of the ligamentous structure of the vertebral column would result in differences in physiological spinal curvatures, based primarily on sacral slope (SS) and pelvic incidence (PI) as key reference indicators. This categorization of the normal sagittal vertebral column morphology into four types is commonly known as the Roussouly classification [3]. An increasing number of surgeons are focusing on the impact of lumbar vertebral soft tissue structure on lumbar degeneration. In 2017, the European Spine Society proposed the Global Alignment and Proportion (GAP) score. This score calculates the overall condition of spinal soft tissues based on the PI as the primary parameter and predicts the risk of postoperative mechanical complications [4]. Since the initial publication of the GAP score, multiple groups have corroborated its ability to predict mechanical complications. However, there are conflicting findings on its accuracy in clinical application, and there is considerable controversy in its assessment. Some groups have demonstrated a strong correlation between the GAP score and predicting clinical and radiographic outcomes, and others have reported weak predictive value [5–12]. Therefore, some scholars assert that the GAP score’s predictive model may inadequately encompass risk factors for postoperative mechanical failure [13].

The assessment of posterior soft tissues using the GAP score only includes ligamentous structures, disregarding the degenerative condition of the paraspinal muscles. The paraspinal muscles are essential for maintaining the stability of the sagittal position of the spine, and their degeneration is currently regarded as closely associated with several lumbar diseases [14, 15]. The degeneration of paravertebral muscles is primarily characterized by atrophy and fat infiltration, leading to diminished strength and contributing to a vicious cycle of degenerative deformation of the lumbar spine [16]. Recent literature confirms that back muscle degeneration occurs earlier than the reduction in lumbar lordosis, and the degeneration of back muscles accelerates the overall degeneration of the lumbar spine, ultimately leading to imbalance in the sagittal position of the spine [17].

Our prior study involved a correlation analysis between the paraspinal muscles and the spinal pelvic parameters, revealing that back muscle degeneration correlates with sagittal position deformity of the spine, especially overall sagittal imbalance and reduced lumbar lordosis [16]. However, spinal pelvic parameters do not reflect the condition of spinal soft tissues. We hope to find a more comprehensive assessment method; however, there are currently no studies investigating the correlation between the GAP score and the degeneration of paravertebral muscles. Therefore, we hope to find the correlation between them and gain a deeper understanding of which assessment method exhibits a stronger correlation with the back muscles by comparing the GAP score to the spinal pelvic parameters. Herein, we aimed to elucidate the relationship between the functional cross-sectional area (FCSA) and fatty infiltration (FI) of the paraspinal muscles with the GAP score using imaging evaluation.

## Materials and methods

### Participants

This study included patients who underwent radiographic assessment and paravertebral muscle endurance testing at Southwest Hospital, Army Medical University (Chongqing, China), between February 2022 and November 2023. The study was approved by the Ethics Committee of Southwest Hospital of Army Medical University (Approval Letter No. KY2020235), and all participants provided written consent before participating in this study.

The inclusion criteria were as follows: (1) patients aged ≥ 45 years; (2) patients with available complete standing full-length spine radiographs and lumbar spine magnetic resonance imaging (MRI) scans suitable for analysis; (3) patients who could tolerate the paraspinal muscle endurance testing; (4) patients who provided consent for the use of anonymized clinical data for research purposes.

The exclusion criteria included the following: (1) Patients with previous spinal fracture, congenital spinal disease, spinal tumor, spinal tuberculosis, or spinal surgery; (2) patients with severe acute or chronic low back pain; (3) patients with neuromuscular disease; (4) patients with MRI contraindications.

### Radiographic measurements

**Fig. 1:**
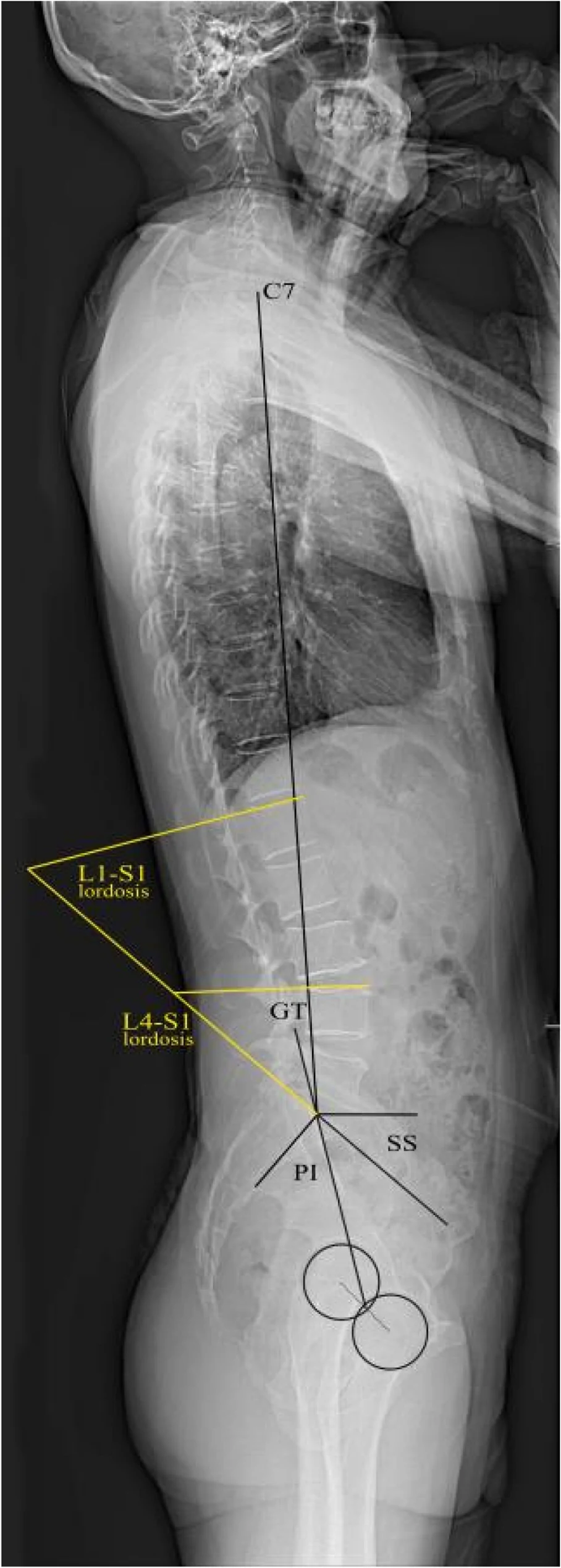
Standing full-length spine radiographs showing the sagittal alignment parameters, including pelvic incidence (PI), sacral slope (SS), and global tilt (GT)

Two orthopedic surgeons independently measured standing full-length spine radiographs using validated software (Picture Archiving and Communication System) (Fig. 1). The following sagittal parameters were measured: (1) pelvic incidence (PI); (2) sacral slope (SS); (3) L1-S1 lumbar lordosis (LL); (4) L4-S1 LL; (5) global tilt (GT).

### Calculation of GAP Score

**Fig. 2:**
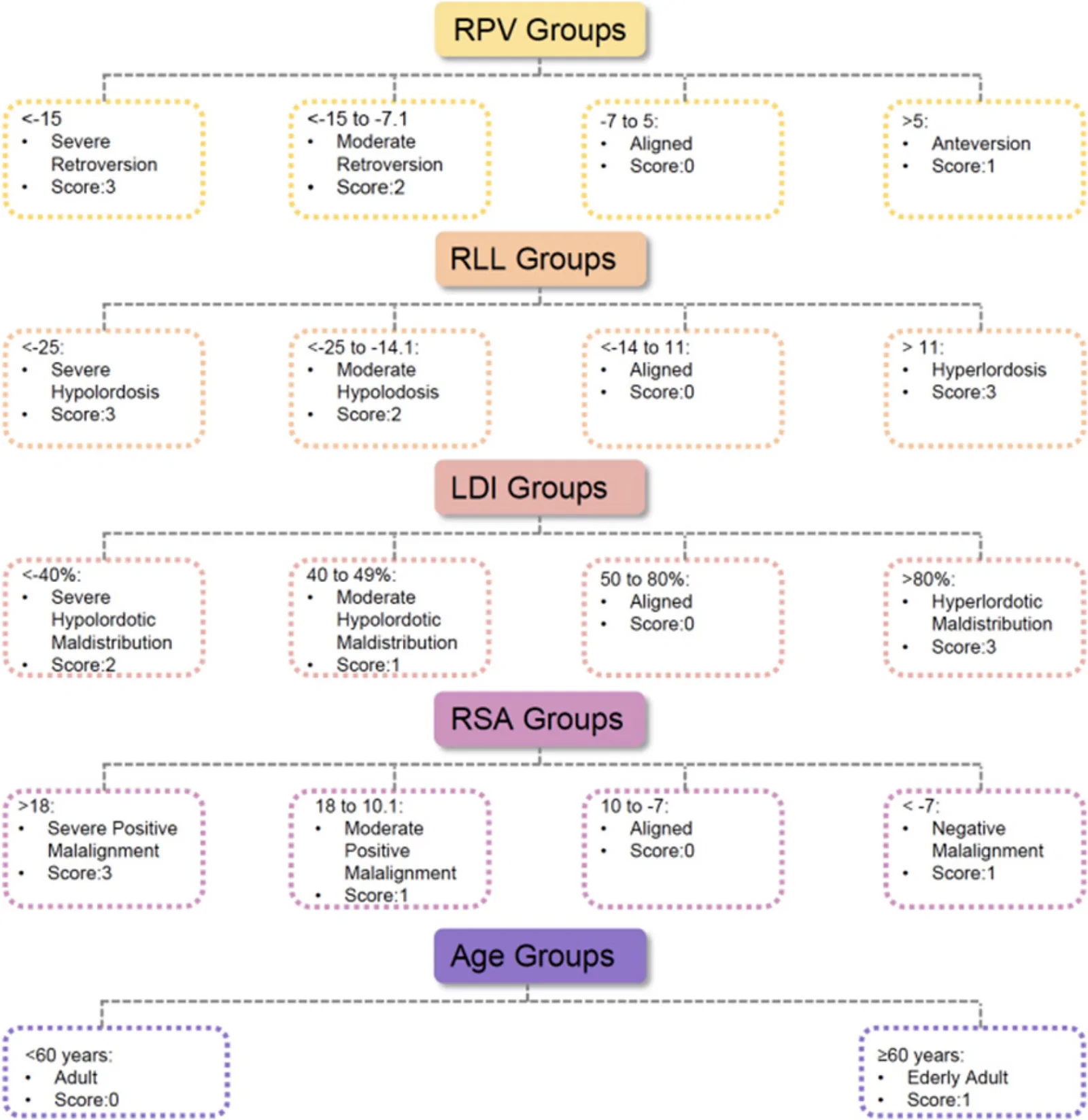
Each component of the GAP score includes a value based on the radiographic parameters and an age component, as depicted in Fig. 2. RPV, relative pelvic version; RLL, relative lumbar lordosis; LDI, lumbar distribution index; RSA, relative spinopelvic alignment

The GAP score was calculated according to the formula of Yilgor et al.: ideal SS = (0.59 × PI + 9), ideal LL = (0.62 × PI + 29), and ideal GT = (0.48 × PI-15)(Fig. 2) [4]. The GAP score ranged from 0 to 13 points. A GAP score of 0–2 indicated a proportioned spinopelvic state; 3–6 indicated a moderately disproportioned state; and $\:\ge\:$7 indiacted a severely disproportioned state.

### MRI paraspinal muscle evaluation

All participants underwent MRI examination using the same equipment (Siemens Healthineers, 3.0 Tesla Imaging System). The repetition duration and echo time were 644 and 9.5 ms, respectively, for T1-weighted imaging, and 4,290 and 93 ms for T2-weighted imaging. The slice thickness was 3 mm, the field of view was 240 × 240 mm, and each layer was a 512 × 384 matrix. The multifidus (MF) and erector spinae (ES) were measured bilaterally from T1-weighted imaging and T2-weighted imaging at the disc level from L4.

**Fig. 3:**
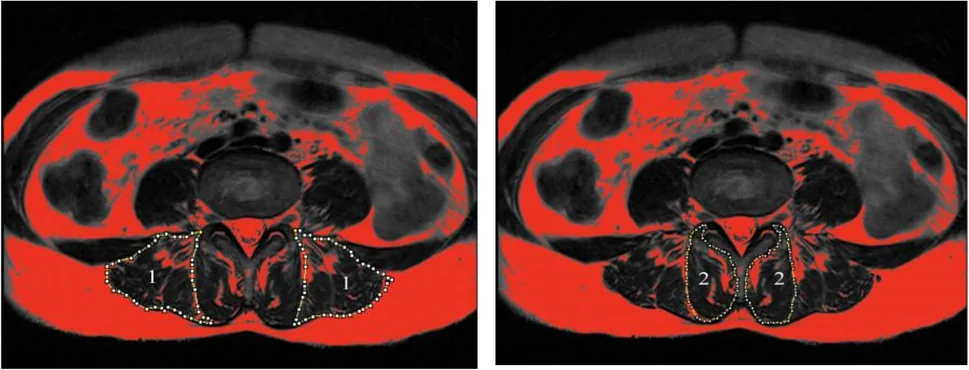
Axial MRI depicting the L4 level paraspinal muscle, where red indicates areas of fat infiltration identified using threshold techniques. (1) Erector spinae and (2) multifidus

The following parameters were measured using ImageJ software (version 2.1.4.7; National Institutes of Health): total cross-sectional area (TCSA, including functional muscle and intramuscular fat), FCSA (the area of lean muscle tissue), and FI (the ratio of TCSA minus FCSA to TCSA), which were measured using the previously reported thresholding technique (Fig. 3) [18]. These findings indicated the degeneration of the paraspinal muscles. All criteria were assessed independently by two orthopedic surgeons. The mean values of their results were utilized.

### Statistical analysis

The Statistical Package for the Social Sciences software (Version 26.0) was used for statistical analysis. After the GAP score was calculated, the patients were categorized into three groups: proportioned (P), 0–2 points; moderately disproportioned (MD), 3–6 points; and severely disproportioned (SD), 7–13 points. The results are presented as the mean ± standard deviation for continuous variables and n(%) for categorical variables. The Shapiro–Wilk test was employed to evaluate the normality of continuous data. Data conforming to a normal distribution are presented as mean ± standard deviation and were analyzed using parametric tests (Pearson correlation, one-way ANOVA). Data that significantly deviated from normality are presented as median (interquartile range) and were analyzed using Spearman correlation. Categorical data are presented as n(%) and were analyzed using the chi-square test. A *P* < 0.05 was considered statistically significant.

## Results

### General information

We identified 61 patients who met the inclusion criteria; 20 patients were in the proportioned group, 14 patients were in the moderately disproportioned group, and 27 patients were in the severely disproportioned group. No significant difference was observed in age, gender, right and left hand grip strength, smoking, alcohol consumption, hypertension, and diabetes prevalence among the three groups (*P* > 0.05). There was a statistical difference in body mass index among the three groups (*P* = 0.044) (Table 1).

**Table 1:** Demographic characteristics of the three groups (means ± SD)

Items	P Group(*n* = 20)	MD Group(*n* = 14)	SD Group(*n* = 27)
Age (years)	59.6 ± 7.081	61.79 ± 6.015	64.41 ± 7.727
Gender			
male	2(10%)	5(35.7%)	4(14.8%)
female	18 (90%)	9(64.3%)	23(85.2%)
BMI (kg/m^2^)	23.15 ± 2.43	25.19 ± 3.12	24.68 ± 2.25
HGS			
R	26.69 ± 5.81	29.07 ± 7.79	23.34 ± 7.85
L	25.23 ± 6.43	28.86 ± 7.05	23.70 ± 7.51
Smoking			
No	20 (100%)	9(64.3%)	23(85.2%)
Yes	0 (0%)	5 (35.7%)	4(14.8%)
Regular Alcohol Intake			
No	18 (90%)	9 (64.3%)	21 (77.8%)
Yes	2 (10%)	5 (35.7%)	6 (22.2%)
Hypertension			
No	16(80%)	12(85.7%)	21(77.8%)
Yes	4(20%)	2(14.3%)	6(22.2%)
Diabetes			
No	19(95%)	13(92.9%)	26(95.1%)
Yes	1(5%)	1(7.1%)	1(3.7%)

*SD* standard deviation, *BMI* body mass index, *P* proportioned, *MD* moderately disproportioned, *SD* severely disproportioned, *HGS* hand grip strength, *R* right, *L* left

### Comparison of paraspinal muscles among the three groups

**Fig. 4:**
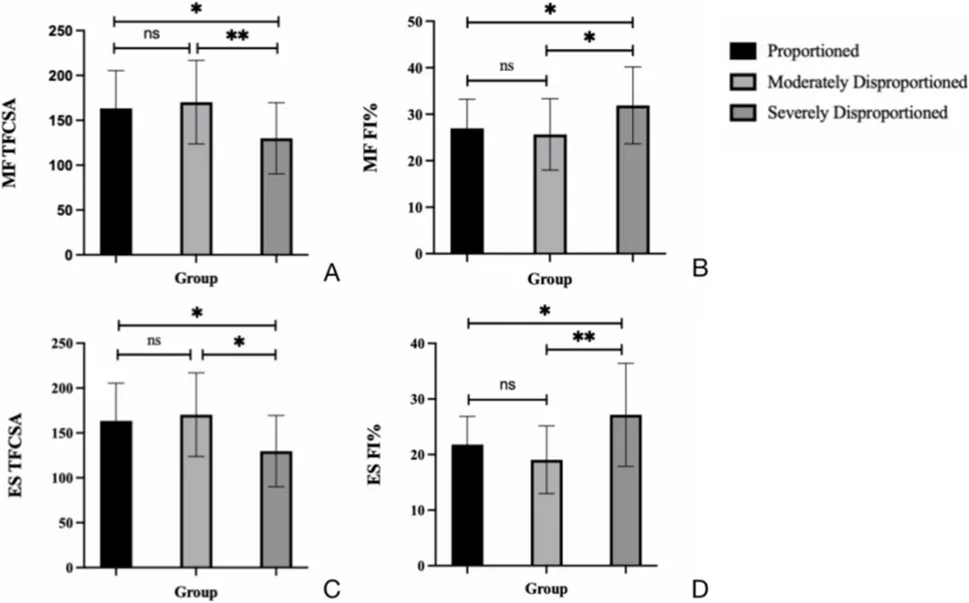
Differences in the paraspinal muscles among the three groups. **A** Multifidus total functional cross-sectional area, **B** multifidus FI, **C** erector spinae total functional cross-sectional area, and **D** erector spinae fatty infiltration. ns, no significant, *P < 0.05, **P < 0.01

To investigate the differences in the paraspinal muscles, we assessed the parameters of the multifidus and erector spinae muscles, including FCSA (R/L), TFCSA, and FI. The FI of MF and ES in the SD group was significantly higher than those in MD groups (mean difference: 0.062, 95% confidence interval [CI]: 0.013 to 0.112, *P* < 0.05 for MF; mean difference: 0.081, 95% CI: 0.032 to 0.130, *P* < 0.05 for ES) and P groups (mean difference: 0.049%, 95% CI: 0.005 to 0.094, *P* < 0.05 for MF; mean difference: 0.054, 95% CI: 0.010 to 0.098, *P* < 0.05 for ES), whereas no significant difference was observed between P and MD groups (*P* > 0.05). The TFCSA of MF and ES in the SD group were significantly lower than those in MD (mean difference: − 236.3, 95% CI: − 392.13 to − 80.47, *P* < 0.05 for MF; mean difference: − 475.84, 95% CI: − 763.0 to − 188.68, *P* < 0.05 for ES) and P groups (mean difference: − 157.32, 95% CI: − 296.91 to − 17.72, *P* < 0.05 for MF; mean difference: − 284.57, 95% CI: − 27.34 to − 541.81, *P* < 0.05 for ES), whereas no significant difference was observed between P and MD groups (*P* > 0.05). The FCSA (R) of MF and ES in the SD group were significantly lower than those in MD and P groups (*P* < 0.05). The FCSA (L) of the MF in the SD group was smaller than that in MD and P groups (*P* < 0.05), whereas the FCSA (L) of the ES in the P group was not significantly different in MD and SD groups (*P* > 0.05) (Table 2; Fig. 4).

**Table 2:** Paraspinal muscle parameters of the three groups (mean ± SD)

Items	P Group(*n* = 20)	MD Group(*n* = 14)	SD Group(*n* = 27)	*P* value	*P* VS. MD	*P* VS. SD	MD VS. SD
MF FCSA (R)	560.53 ± 113.25	612.10 ± 132.61	476.34 ± 137.00	0.005	0.255	0.031	0.002
MF FCSA (L)	574.25 ± 92.88	601.67 ± 140.98	501.12 ± 128.07	0.026	0.518	0.045	0.014
MF TFCSA	1134.78 ± 196.77	1213.77 ± 260.45	977.47 ± 249.86	0.008	0.342	0.028	0.004
MF FI	26.97 ± 6.27	25.66 ± 7.69	31.91 ± 8.26	0.022	0.621	0.030	0.015
ES FCSA (R)	985.66 ± 167.02	1025.58 ± 272.58	805.30 ± 249.20	0.006	0.623	0.011	0.005
ES FCSA (L)	950.08 ± 135.04	1101.42 ± 276.44	845.87 ± 267.14	0.007	0.208	0.242	0.026
ES TFCSA	1935.74 ± 266.90	2127.00 ± 536.34	1651.17 ± 476.83	0.004	0.551	0.038	0.030
ES FI	21.80 ± 5.09	19.06 ± 6.09	27.15 ± 9.29	0.003	0.297	0.018	0.002

*MF* multifidus, *ES* erector spinae, *FI* fatty infiltration, *FCSA* functional cross-sectional area, *TFCSA* total functional cross-sectional area, *P* proportioned, *MD* moderately disproportioned, *SD* severely disproportioned

Relative signal intensity * indicates a *P* value < 0.05; ** indicates a *P* value < 0.01; *** indicates a *P* value < 0.001

### Correlation between GAP score and paraspinal muscles

**Fig. 5:**
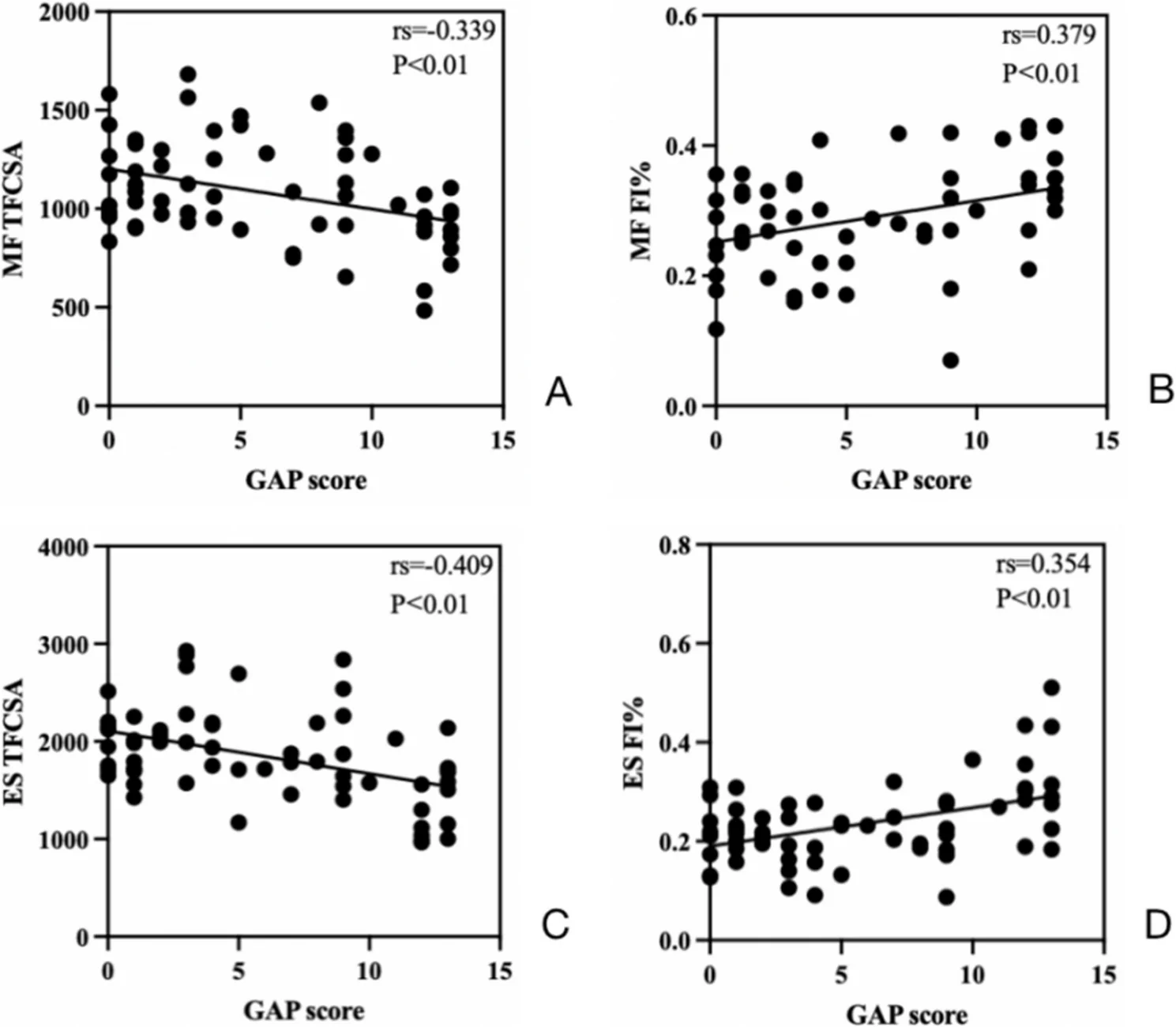
Correlations between GAP score and (**A**) multifidus total functional cross-sectional area, (**B**) multifidus fatty infiltration, (**C**) erector spinae total functional cross-sectional area, and (**D**) erector spinae fatty infiltration

No significant negative correlation was observed between MF TFCSA, ES TFCSA, and GAP scores (*r* = − 0.339, − 0.409, *P* < 0.01). Additionally, a significant positive correlation was observed between MF FI, ES FI, and GAP score (*r* = 0.379, 0.354, *P* < 0.01) (Table 3; Fig. 5).

**Table 3:** Correlation analysis between the GAP score and paraspinal muscle parameters

Variables	MF TFCSA	MF FI	ES TFCSA	ES FI
GAP score				
R	-0.339	0.379	-0.409	0.354
P	0.007	0.003	0.001	0.005

*R* Spearman correlation coefficient, *MF TFCSA* multifidus total functional cross-sectional area, *MF FI* multifidus fatty infiltration, *ES TFCSA* erector spinae total functional cross-sectional area, *ES FI* erector spinae fatty infiltration

### Correlations between the paraspinal muscles and GAP score-related parameters (Fig. 6).

**Fig. 6:**
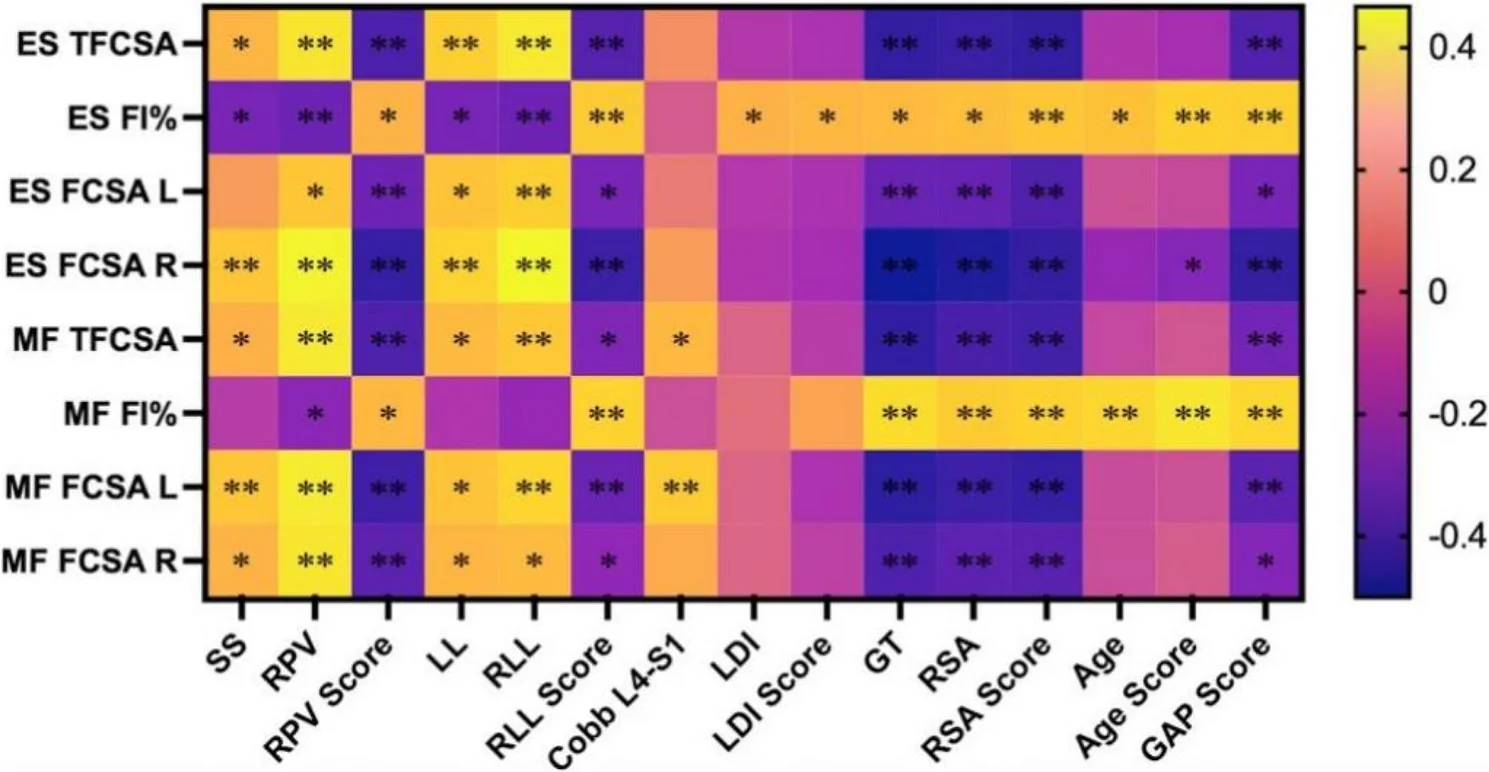
Correlation coefficient r; yellow indicates a positive correlation, and blue indicates a negative correlation. MF, multifidus; ES, erector spinae; FI, fatty infiltration; FCSA, functional cross-sectional area; TFCSA, total functional cross-sectional area; R, right; L, left; SS, sacral slope; RPV, relative pelvic version; LL, lumbar lordosis; RLL, relative lumbar lordosis; LDI, lordosis distribution index; GT, global tilt; RSA, relative spinopelvic alignment. * indicates a *P* < 0.05; ** indicates a *P* < 0.01

## Discussion

The GAP score predominantly uses the PI as the main reference index to obtain the ideal values of the spinal pelvic parameters for each patient. The scores of the various indices are calculated by comparing the ideal values with the measured values. This study suggested that an increase in the score correlates with the deterioration in spinal imbalance, resulting in a higher occurrence of postoperative mechanical complications [6]. However, there is significant controversy regarding its predictive function in clinical practice [11]. The GAP score was initially developed because the current classification and grading methods for adult spinal deformities were inadequate, hindering proper surgical guidance and the prediction of postoperative mechanical complications [4]. With advancements in spinal surgery, spinal surgeons have recognized that most spinal evaluation criteria primarily rely on full-length spinal X-rays and bone morphology as reference standards, without giving much attention to spinal soft tissue structure. However, the spinal soft tissue structure undergoes significant alterations with age, which can significantly affect spinal morphology and function [19].

Our correlation analysis revealed a negative correlation between the FCSA of multifidus and erector spinae muscles and the GAP score (*r* = − 0.339, − 0.409, *P* < 0.01), and FI was positively correlated (*r* = 0.379, 0.354, *P* < 0.01). A greater severity of paraspinal muscle degeneration corresponded to an increased GAP score. Previous studies have confirmed the significance of the paravertebral muscle in predicting postoperative complications; hence, adding paravertebral muscle parameters may enhance the predictive ability of the GAP score and yield a more comprehensive assessment of spinal mechanics and balance [20, 21].

The GAP score introduces the theory of the “core cone,” which asserts that when a person is standing, the body is most stable when it is perpendicular to the ground from the head to the feet. Core muscle groups are essential for stabilizing the trunk [22]. In upright posture, the energy expenditure of core muscle groups is minimal, but increases during forward flexion or backward extension [4, 22]. Previous studies demonstrated that core muscle groups, including the abdominal muscles and the erector spinae, degenerate slowly, whereas the back muscles, particularly the paravertebral muscles, are the primary muscle groups responsible for sustaining trunk stability [2, 23]. Furthermore, their degeneration becomes increasingly evident with age, primarily due to atrophy and FI, resulting in diminished muscular strength [24]. In the intergroup comparison, the FCSA of the paravertebral muscles in the SD group was significantly smaller than that in the other two groups, and the FI was significantly greater in this group. No significant differences were observed between P and MD groups. Consequently, the weakening of the paravertebral muscles due to degeneration is one of the reasons for severe spinal imbalance.

Lenke. et al. examined the overall sagittal alignment of 85 volunteers utilizing EOS technology and performed a correlation study with the GAP scores. The proportion of healthy volunteers with moderate and severe imbalance after the age of 60 significantly increased, with no participant under 60 years old in the severe imbalance group. Although the article lacks relevant parameters for the paravertebral muscles, it confirms that degeneration of the paravertebral muscles can result in increased spinal imbalance with advancing age [19]. Recent literature has delineated different segments of sagittal spinal balance, which are divided into four stages: sagittal balance (SB), compensated sagittal balance (CSB), decompensated sagittal balance (DSI), and sagittal balance with failure of polar compensation (SIFPC) [17]. The classification primarily pertains to the reduction in lordosis during lumbar degeneration, resulting in pelvic rotation compensation, which leads to decompensation [25]. One of the special stages (SIFPC) is the overall sagittal decompensation of the spine, which occurs before significant compensation for pelvic rotation occurs in the reduction of lumbar lordosis [26, 27]. Li Weishi et al. published an article analyzing the correlation with the paravertebral muscles. Analysis revealed that the primary issue with sagittal spinal balance is the degeneration of the paravertebral muscles, resulting in diminished strength and further activation of the pelvic compensation mechanism. The study specifically states that early and severe degeneration of the paravertebral muscles can initiate the SIFPC stage, indicating that the pelvic compensatory mechanism has not commenced, leading to a general sagittal imbalance of the spine [17]. Based on the GAP score and our analysis, soft tissue degeneration in the lower back is one of the initiating factors for the onset of spinal degeneration.

Besides the age dimension, the GAP score comprises four main dimensions: RPV, RLL, LDI, and RSA. This study demonstrated that relative sagittal position parameters were more strongly correlated with the lumbar and back muscles compared to conventional sagittal spinal pelvic parameters (SS, LL, and GT). Conventional spinal pelvic parameters rely on full-length spinal X-rays, focusing primarily on bone evaluation criteria, resulting in various classification systems [28]. A notable example is the Scoliosis Research Society-Schwab classification, employed to guide surgical treatment for degenerative spinal deformities. However, many issues have been identified in its application, despite later revisions integrating age factors [29]. The emergence of the Roussouly classification and the identification of varying lumbar and back ligament structures leading to different spinal sagittal morphologies have revealed that PI exhibits considerable variability, ranging from 27° to 50°. The sagittal balance of the spine in the normal population has been characterized based on the PI and other criteria, highlighting the significance of the lower lumbar curvature on sagittal spinal balance [3]. The GAP score was developed based on these findings, emphasizing the spinal ligament and soft tissue structures. However, the GAP score overlooks the significance of the lumbar and back muscles, and although the assessment of lumbar and back muscle function remains underdeveloped, its significance is gradually being recognized.

This study has several limitations. First, this was only a single-center retrospective study, which might introduce selection bias. Second, the generalizability of our findings may be limited by the gender imbalance in our cohort, which exhibited a predominance of female participants. Third, although our overall sample size achieved the pre-established aim, the distribution of patients into three GAP categories resulted in modest subgroup sizes. This may affect the reliability of some subgroup comparisons. Causality cannot be confirmed. Future prospective studies necessitating the recruitment of more participants with a balanced gender distribution are essential to confirm our conclusions.

## Conclusion

The extent of paraspinal muscle degeneration was closely associated with the GAP score. The severity of paraspinal muscle degeneration correlated positively with the GAP score. The relative sagittal plane parameters (RPV, RLL, and RSA) exhibited a stronger correlation with paraspinal muscle parameters than with actual spinal pelvic parameters, thereby more accurately reflecting the degeneration of paraspinal muscle and offering a more comprehensive assessment of spinal mechanics and balance. However, it is crucial to interpret these findings with caution due to the study’s limitations, which encompass a small sample size and gender imbalance.

## Data Availability

The datasets used and/or analyzed during the current study are available from the corresponding author on reasonable request.
